# Development and validation of the Italian version of the Mobile Application Rating Scale and its generalisability to apps targeting primary prevention

**DOI:** 10.1186/s12911-016-0323-2

**Published:** 2016-07-07

**Authors:** Alexander Domnich, Lucia Arata, Daniela Amicizia, Alessio Signori, Bernard Patrick, Stoyan Stoyanov, Leanne Hides, Roberto Gasparini, Donatella Panatto

**Affiliations:** Department of Health Sciences, University of Genoa, Genoa, Italy; School of Medical and Pharmaceutical Sciences, University of Genoa, Genoa, Italy; Institute of Health & Biomedical Innovation, School of Psychology and Counselling, Queensland University of Technology, Brisbane, Australia; The Young and Well Cooperative Research Centre, Abbotsford, Australia

**Keywords:** Mobile health, mHealth, Mobile application, Mobile application rating scale, Prevention

## Abstract

**Background:**

A growing body of literature affirms the usefulness of mobile technologies, including mobile applications (apps), in the primary prevention field. The quality of health apps, which today number in the thousands, is a crucial parameter, as it may affect health-related decision-making and outcomes among app end-users. The mobile application rating scale (MARS) has recently been developed to evaluate the quality of such apps, and has shown good psychometric properties. Since there is no standardised tool for assessing the apps available in Italian app stores, the present study developed and validated an Italian version of MARS in apps targeting primary prevention.

**Methods:**

The original 23-item version of the MARS assesses mobile app quality in four objective quality dimensions (engagement, functionality, aesthetics, information) and one subjective dimension. Validation of this tool involved several steps; the universalist approach to achieving equivalence was adopted. Following two backward translations, a reconciled Italian version of MARS was produced and compared with the original scale. On the basis of sample size estimation, 48 apps from three major app stores were downloaded; the first 5 were used for piloting, while the remaining 43 were used in the main study in order to assess the psychometric properties of the scale. The apps were assessed by two raters, each working independently. The psychometric properties of the final version of the scale was assessed including the inter-rater reliability, internal consistency, convergent, divergent and concurrent validities.

**Results:**

The intralingual equivalence of the Italian version of the MARS was confirmed by the authors of the original scale. A total of 43 apps targeting primary prevention were tested. The MARS displayed acceptable psychometric properties. The MARS total score showed an excellent level of both inter-rater agreement (intra-class correlation coefficient of .96) and internal consistency (Cronbach’s *α* of .90 and .91 for the two raters, respectively). Other types of validity, including convergent, divergent, discriminative, known-groups and scalability, were also established.

**Conclusions:**

The Italian version of MARS is a valid and reliable tool for assessing the health-related primary prevention apps available in Italian app stores.

**Electronic supplementary material:**

The online version of this article (doi:10.1186/s12911-016-0323-2) contains supplementary material, which is available to authorized users.

## Background

Primary prevention is an essential multidisciplinary approach to reducing both mortality and morbidity [1] and has proved to be highly effective in reducing the burden of several communicable and non-communicable diseases [2, 3]. However, the traditional methods of enhancing primary prevention may produce only modest benefits, especially in population groups that are difficult to reach and engage, such as teenagers and immigrants [4]. The implementation of information technologies (ITs) in the field of preventive medicine may help to overcome these limitations, and is now becoming increasingly common. A particularly attractive and promising means of delivering public health interventions is mobile technologies, since mobile devices are almost universally adopted, easily portable and endowed with increasingly sophisticated technical features [5]. Moreover, some evidence of the efficacy of mobile health (mHealth) preventive interventions, including text messaging [6, 7] and mobile apps [8–10], has already been established.

There are now more than 100,000 health-related mobile apps [11]; an average smartphone owner has 41 apps installed and 19 % of owners use health-related apps [12]. Despite these huge numbers, more than half of health-related apps have few (<500) downloads [13], a quarter of installed apps are never used [14] and many are non-evidence-based or of low quality [15]. This last issue is of particular importance, since inaccurate, misleading or out-of-date information may impair health-related decision-making and outcomes [16]. Apart from the information domain, other attributes of the quality (e.g. user-friendliness and -satisfaction, usability and aesthetics) of apps need to be considered [17].

The Mobile Application Rating Scale (MARS) [18] was recently developed to address these issues. The 23-item scale assesses the quality of health-related apps on four objective quality dimensions (engagement, functionality, aesthetics and information) and one subjective dimension. The original version of the tool has demonstrated high levels of reliability in rating the quality of mental health-related apps available in the Australian Apple Store [18]. More recently, the usefulness of applying MARS to evaluating the quality of mindfulness-based [19] and weight-loss and smoking cessation [20] apps has been demonstrated.

There is currently no instrument for assessing the rapidly growing number of health-related mobile apps available in Italian app stores. The present study aimed to develop and test the reliability and validity of an Italian version of MARS. Arguably, the original English version of MARS could be successfully used to evaluate apps in Italy if users were proficient in the English language. However, only 26 % of Italians are able to read newspapers or magazines in English [21]. Moreover, proficiency in English is unsatisfactory even among Italian physicians and specialists; 47.9 % rate their knowledge of English as very low or low, while only 21.6 % claim to have good/excellent English language skills [22]. The potential impact of the present study is twofold. First, it could provide researchers and public health professionals with a standardised tool for assessing qualitative aspects of the growing number of mHealth apps available in Italian app stores. Second, it demonstrates the generalisability of MARS to apps targeting preventive medicine.

## Methods

### Study design

This validation study applied the well-established process of cross-cultural adaptation [23], translation and back-translation, review, piloting, and psychometric evaluation.

### Mobile application rating scale (MARS)

The initial development and evaluation of the psychometric properties of the English version of MARS have been reported elsewhere [18]. Briefly, the tool consists of a description/classification section and 23 items rated on an anchored 5-point Likert-type response scale, assessing app quality on four objective quality dimensions/subscales: engagement (5 items), functionality (4 items), aesthetics (3 items), information (7 items), as well as a fifth subjective quality dimension (4 items). Several items (items 14–17, 19) in the information subscale have a “Not applicable” (N/A) response option. Scores of individual items are averaged to obtain a mean quality score for each dimension; in turn, the scores on the four objective quality dimensions are averaged to yield a MARS total score. All mean quality scores are on a 1 (poor) to 5 (excellent) scale. The MARS also contains an app-specific section (6 items) to evaluate the potential impact of a particular app on users’ knowledge, intentions etc. According to the authors, the app-specific section and the items on description/classification can be adjusted to research aims. Both the MARS total and subscale scores have very high Cronbach’s alpha (*α*) coefficients (.90 and .80–.89, respectively) and acceptable levels of inter-rater reliability and agreement and convergent validity with app-store star ratings.

### Adaptation and translation processes

The linguistic validation of MARS involved several steps. Since the English version of MARS contains numerous IT terms, most of which have recently been taken up by the Italian language as loanwords with different degrees of integration, we decided to proceed with adaptation of the scale to Italian. The adaptation process adopted the universalist approach, as described by Herdman et al. [23]. In their model, these authors distinguish six types of equivalence (conceptual, item, semantic, operational, measurement and functional) and provide their definitions, methods of evaluation and possible outcomes. Before the translation itself was undertaken, the conceptual and item equivalences were verified empirically; these were examined through a review of local IT literature (in particular, on usability) and consultation with IT specialists. Once the conceptual and item equivalences had been demonstrated, two independent translations were conducted by two native Italian speakers with different educational backgrounds (a medical doctor and an IT specialist) and good English proficiency. To facilitate the achievement of semantic equivalence, a clear understanding of the wording of items and response options was acquired by the research staff; if necessary, the corresponding author of the original publication was contacted for clarification. Following the review and discussion of the two forward translations, a reconciled version was produced. This latter underwent blind backward translation by a bilingual native English speaker (BP) and was subsequently compared with the original version.

### Selection of apps

The development and application of a systematic search strategy for directly identifying apps in app stores may be challenging for at least three reasons. First, app stores use ranking algorithms, with the result that more popular apps often appear before those that are more appropriate to entry terms. Second, the use of specific entry terms may yield irrelevant results, since the indexing of apps is usually determined by a developer who is interested in promoting the app, and thus often includes broader index terms. Third, there is no way of searching for apps simultaneously in several app stores [24]. In the present study, in order to mitigate these methodological challenges, apps were sought in three app stores (Google Play, Apple and Windows Stores) on 6^th^ August 2015 by means of a method, albeit not fully systematic, similar to that described by Schnall and Iribarren [25]. The following terms were searched for in each app store: *prevention*, *prevent*, *prophylaxis*, *risk factor*, *risk factors*, *risk*, *risks*, *vaccination*, *vaccinations*, *vaccine*, *vaccines*, *health education*. Entering more detailed search terms (e.g. disease prevention) yielded only duplicates and did not increase specificity. The following inclusion criteria were applied: (i) app availability in the Italian language (from the app description in the app stores) and (ii) relevance (at least partial) to primary prevention (e.g. vaccination, health promotion, health education, identification of risk factors etc.). Exclusion criteria were: (i) no Italian version; (ii) irrelevance to primary prevention, or primary prevention by means of alternative medicine, or focus only on fitness, physical exercise or calorie counts/diets; and (iii) medical apps targeting only health professionals.

### Assessment of apps

To evaluate apps, we roughly followed the methodology of the original study [18]. Specifically, as recommended by Stoyanov et al. [18], two raters (AD and LA) attended and discussed three MARS training modules freely available on YouTube. Both raters developed a shared strategy for understanding of the app target groups. Subsequently, apps that met the inclusion criteria were downloaded to iPhone 5, Huawei P8 and Nokia Lumia 520 devices from the three app stores. For the purpose of piloting, 5 apps were first tested for at least 10 min, and the pre-final Italian version of MARS was then applied in order to rate their quality; this step was carried out by the two raters, each working independently. The raters then compared their scores on each of the 5 apps and any disagreements were discussed until consensus ratings were achieved. Psychometric properties of apps included in the main study were assessed independently for the two raters.

### Data analysis

The minimum sample size was determined on the basis of previous research [18, 26]. A total of 41 apps were needed in order to establish whether true inter-rater reliability (2 raters) lay within .15 of a sample observation of .80 (empirical assurance of 87 %).

The distribution of summary scores (i.e. 5 subscale scores and the MARS total score) was visually inspected and skewness coefficients were calculated; their normal distribution was formally confirmed by means of the D’Agostino test [27]. Normally distributed data were expressed as means with standard deviations (SDs). Paired *t* tests were used to test the null hypothesis on between-rater equality in summary scores. Floor and ceiling effects of the summary scores were deemed to be present if 15 % of apps had the lowest or highest possible scores, respectively [28].

As in the source study [18], the inter-rater reliability of items, subscales and MARS total scores was measured by means of intra-class correlation coefficients (ICCs) using two-way mixed effects, average measures model with absolute agreement. ICCs were interpreted as excellent (≥ .90), good (.76–.89), moderate (.51–.75) and poor (≤ .50) [29].

Cronbach’s *α* coefficients were computed in order to evaluate internal consistency of the summary scores. Alphas were interpreted as excellent (≥ .90), good (.80–.89), acceptable (.70–.79), questionable (.60–.69), poor (.50–.59) and unacceptable (<. 50) [30]. Additionally, split-half reliability was assessed by means of the Spearman-Brown prophecy formula [31, 32]. This formula was also applied in order to predict the internal consistency of the average of the 2 raters.

Item-total and item-subscale correlations were investigated to establish the convergent validity. Inter-item correlations were also calculated. Specifically, the correlation between two normally distributed continuous variables was quantified by means of Pearson’s *r* coefficients with 95 % confidence intervals (CIs), while correlations between ordinal (integer scores of single Likert-type items) or ordinal and continuous variables were quantified by means of Spearman’s *ρ* coefficients. Item-total and mean inter-item correlation coefficients > .2 and > .3, respectively, were regarded as satisfactory [33]. Between-subscale correlations were calculated in order to evaluate whether pairs of subscales measured unrelated constructs [34]; pairwise coefficients > .7 were considered to be unacceptable. To assess divergent validity, we compared correlation coefficients between an item and its own subscale versus that item and other subscales; the divergent validity was considered satisfactory when > 80 % of item-own subscale correlation coefficients were higher than item-other subscale coefficients [35].

Ferguson's *δ* coefficients were computed to check the ability of each of the summary scores to discriminate among single apps. A scale has maximal discriminating properties (*δ* = 1) when all possible scores occur equally frequently [36]. Loevinger’s *H* coefficient was used to estimate scalability of the summary scores, which may be interpreted as the degree of accuracy with which single items within a subscale are able to order apps (i.e. unidimensionality). Conventionally, *H* coefficients of .30–.39, .40–.49 and ≥ .50 indicate weak, moderate and strong scales, respectively, while those below .30 are not regarded as unidimensional [37].

The methods described by Stoyanov et al. [18] were utilised to establish the concurrent validity of the Italian version of the MARS. Correlations between the MARS star rating (item 23), subjective quality subscale and total score with the app-store star ratings were examined. The concurrent validity of apps with at least 1, 5 and 10 ratings (votes) in the app stores was studied separately; the 5-vote cut-off was used in the source study [18] and 1- and 10-vote cut-offs were introduced empirically in order to verify the robustness of correlation coefficients. As websites developed by government agencies and universities have generally been found to be of higher quality than other sites (commercial, private, etc.) [38, 39] the impact of the affiliation of the app developer on the MARS total score (known-groups validity) was also examined. Differences in the MARS total score of apps with different affiliations (unknown/commercial versus governmental/non-profit organization/university) and the corresponding effect sizes were established by means of *t* test and Cohen’s *d*, respectively; the latter was interpreted as: small (*d* = 0.2), medium (*d* = 0.5) or large (*d* = 0.8) [40].

To formally establish measurement equivalence [23], we compared the Italian version of MARS with the original version in terms of internal consistency (by comparing two independent Cronbach’s *α*s, as described by Charter and Feldt [41]), inter-rater reliability (by examining 95 % CIs) and concurrent validity (by comparing *r* correlation coefficients via Fisher’s *r*-to-*z* transformation). Finally, multivariable linear regression was used to predict the MARS total score from a set of independent variables [app store, app store star rating, number of ratings in app store, affiliation, months since the last update, developer’s origin (Italy vs other countries)] that were selected by means of the all-possible-regressions approach by minimizing the corrected Akaike information criterion. Statistical analysis was performed by means of the R stats package, version 3.1.2 [42].

## Results

### Adaptation and translation processes

The construct of the original MARS version was judged to be conceptually equivalent, since all five domains of the scale are highly relevant and appropriate to the mobile apps available in Italian app stores. No item required any major modifications. However, following the production of two forward versions, an issue regarding the translation of IT terminology arose. Although these semantic neologisms are now widely used by IT specialists in Italy, such loanwords may be unfamiliar to people outside the IT field, such as health professionals. Moreover, technical terms related to smartphones (especially gesture commands) are of very recent introduction. To produce a reconciled version of the scale, these technical terms were artificially divided into three categories: calques (e.g. screen – *schermo*), adapted loanwords (e.g. navigation – *navigazione*), and non-adapted loanwords (e.g. swipe). Terms belonging to the first two categories were reported in their commonly used “Italianized” dictionary form. By contrast, non-adapted loanwords were mostly reported by using the original English spelling accompanied by their referential meaning in Italian. During the production of the two forward translations, the authors of the source questionnaire were contacted for clarification of two items.

A backward translation was subsequently produced; having been judged satisfactory by the research staff, this was sent, without modification, to the corresponding author of the original scale. The intralingual equivalence between the original and backward-translated versions was then discussed with the team of researchers of the source tool. In general, the backward-translated version was deemed highly congruent with the original version. Most comments made by the MARS developers concerned shades of meaning of single words (e.g. use of adverbs of degree). However, some other comments highlighted a possible problem of non-equivalence (e.g. “through games” was not judged equivalent to “through gamification”). All these comments were addressed by finding the closest translation. Some small modifications were also made in the description/classification section [by adding options “Windows Phone” and “Public body” to the items on app platform and affiliation, respectively, and by combining the options “CBT – Behavioral (positive events)” and “CBT – Cognitive (thought-challenging)” into a single option “CBT – Cognitive behavioral therapy”]. Since there were no major changes to be made in the reconciled version, a second backward translation was judged to be unnecessary. It was deemed that the scale format, instructions and measurement would not affect the operational equivalence.

### App selection and piloting

A total of 579 apps were retrieved; after the removal of duplicates (*N* = 132), 447 apps were screened. Of these, 398 apps were excluded on the basis of exclusion criteria 1 (no Italian version, *N* = 42), 2 (not relevant to primary prevention, *N* = 348) and 3 (theory targeted apps for healthcare professionals, *N* = 8), respectively. One app available in the Windows Store was not downloadable, owing to unmet technical requirements (Nokia Lumia 520 has no front camera). Thus, 48 apps were included.

The first 5 apps were piloted and the percentage of absolute agreement was computed. This varied substantially according to the subscale; it was at least 60 % for engagement (64 %), information (69 %) and subjective quality (60 %), while it was 50 % for functionality and 27 % for aesthetics. Following comparison and review of the results of the pilot test, both raters repeated and discussed the training course, in order to improve the alignment of app ratings. No modifications to the scale were deemed necessary. We then proceeded to evaluate the reliability and validity of the final Italian version of MARS (Additional file 1).

### Main study

A total of 43 apps were tested in the validation study. Most of these (*N* = 30, 70 %) were for the Android platform (since searches in Google Play were conducted first), while 9 (21 %) and 4 (9 %) apps were downloaded from Apple and Windows Stores, respectively. About half of the apps were affiliated to unknown or commercial developers, while 26, 12 and 12 % were developed through the participation of non-commercial organizations, government/public health authorities and universities, respectively. The median time since the last update was 12.3 (interquartile range: 6.5–20.0) months. Only one app had previously been tested in formal studies (item 19 of the information subscale); this item was therefore excluded from all calculations.

As shown in Table 1, the distribution of all composite scores was approximately symmetric, as no skewness coefficient exceeded |1|. The D’Agostino test confirmed normal distributions of all the summary scores produced by both raters. The subscale scores of the two raters were very close to each other and the between-rater difference did not exceed 10 %, ranging from 0 % (aesthetics) to 7.2 % (subjective quality). Paired *t* test showed no significant differences between the raters’ scores (engagement: *p* = .22; functionality: *p* = .54; aesthetics: *p* = .99; information: *p* = .86; MARS total score: *p* = .41; subjective quality: *p* = .19). The functionality subscale was probably subject to a ceiling effect, as its score exceeded the pre-specified criterion of 15 %.

**Table 1:** Mean scores, distribution and floor and ceiling effects, by rater and subscale

Scale	Skewness		Mean (SD)		Floor effect, %		Ceiling effect, %	
	Rater 1	Rater 2	Rater 1	Rater 2	Rater 1	Rater 2	Rater 1	Rater 2
Engagement	0.39	0.17	2.87 (0.87)	2.96 (0.79)	0	0	2.3	0
Functionality	−0.28	−0.87	4.10 (0.67)	4.15 (0.80)	0	0	18.6	18.6
Aesthetics	−0.67	−0.56	3.34 (0.99)	3.34 (0.94)	7.0	4.7	4.7	2.3
Information^a^	−0.64	−0.34	3.49 (0.80)	3.48 (0.72)	0	0	0	0
MARS total score	−0.36	−0.34	3.45 (0.66)	3.48 (0.66)	0	0	0	0
Subjective quality	0.39	0.51	2.49 (1.17)	2.31 (0.99)	7.0	14.0	2.3	0

^a^ Item 19 was excluded from all calculations because of lack of ratings

The ICCs were deemed excellent for 4 of the 5 subscales and the MARS total mean quality scores and good for the functionality subscale (Table 2). The ICCs of single items varied in a range of .59–.93, with a mean of .82 (SD: .11): estimates of 7, 9 and 6 items were classified as excellent, good and moderate, respectively (Additional file 2: Table S1). The lowest ICCs (.59 and .60) were observed for items 17 (visual information) and 5 (target group), respectively.

**Table 2:** Intra-class correlation coefficients, by subscale

Scale	ICC*	
	Estimate	95 % CI
Engagement	.91	.84–.95
Functionality	.88	.77–.93
Aesthetics	.93	.87–.96
Information^a^	.95	.90–.97
MARS total score	.96	.93–.98
Subjective quality	.95	.89–.97

*All *p* < .001; ^a^Item 19 was excluded from all calculations because of lack of ratings

All Cronbach’s *α* coefficients were judged to be at least acceptable, independently of both rater and subscale. Notably, these were categorized as excellent for the MARS total and subjective quality subscale scores (Table 3). Moreover, the MARS total score displayed relatively stable internal consistency, as shown by the Spearman-Brown prophecy formula (Rater 1: .81; Rater 2: .84). The estimate of the internal consistency of the average of the MARS total scores assigned by the 2 raters was also good (.85).

**Table 3:** Cronbach’s *α* coefficients, by rater and subscale

Subscale	Cronbach’s *α* (95 % CI)	
	Rater 1	Rater 2
Engagement	.85 (.76–.91)	.84 (.75–.90)
Functionality	.77 (.63–.87)	.87 (.79–.92)
Aesthetics	.92 (.86–.95)	.88 (.81–.93)
Information^a^	.73 (.57–.84)	.71 (.54–.83)
MARS total score	.90 (.85–.94)	.91 (.87–.94)
Subjective quality	.95 (.92–.97)	.93 (.89–.96)

^a^Item 19 was excluded from all calculations because of lack of ratings

The convergent validity of the Italian MARS was established, as the item-subscale and item-total correlation coefficients of both raters exceeded the cut-off value of .2; after correction for overlapping, most item-total *ρ*s (16/22 and 18/22 for raters 1 and 2, respectively) were ≥ .5 (Table 4). Some item-total correlation coefficients were not, however, statistically significant (item 14 as measured by both raters and items 13 and 4 as measured by raters 1 and 2, respectively) as shown by the corresponding 95 % CIs. Similarly, as shown by the correlation matrixes (Additional file 2: Figure S1), most *ρ*s were > .2 and the average inter-item correlation coefficient also fulfilled the pre-specified criterion (Rater 1: .40; Rater 2: .43).

**Table 4:** Corrected item-subscale and item-total Spearman’s *ρ* correlation coefficients, by rater

Subscale	Item	Corrected item-subscale correlation, *ρ* (95 % CI)		Corrected item-total correlation, *ρ* (95 % CI)	
		Rater 1	Rater 2	Rater 1	Rater 2
Engagement	1	.80 (.65–.89)	.81 (.68–.89)	.78 (.61–.88)	.74 (.55–.86)
	2	.82 (.64–.92)	.79 (.64–.89)	.78 (.62–.88)	.75 (.57–.86)
	3	.47 (.20–.68)	.71 (.52–.84)	.35 (.06–.59)	.62 (.37–.78)
	4	.62 (.35–.82)	.44 (.15–.69)	.54 (.24–.76)	.28 (−.03–.56)
	5	.61 (.39–.77)	.54 (.28–.74)	.77 (.60–.88)	.69 (.48–.84)
Functionality	6	.64 (.41–.82)	.62 (.40–.76)	.48 (.20–.69)	.42 (.11–.67)
	7	.50 (.22–.72)	.71 (.51–.84)	.33 (.02–.60)	.62 (.38–.79)
	8	.75 (.56–.88)	.78 (.63–.87)	.45 (.17–.68)	.74 (.57–.86)
	9	.65 (.44–.81)	.80 (.62–.90)	.53 (.29–.70)	.73 (.53–.86)
Aesthetics	10	.69 (.45–.84)	.60 (.36–.78)	.82 (.66–.91)	.69 (.50–.83)
	11	.75 (.55–.89)	.88 (.80–.93)	.60 (.35–.78)	.75 (.57–.86)
	12	.86 (.73–.93)	.87 (.76–.93)	.68 (.48–.82)	.75 (.55–.87)
Information^a^	13	.33 (.03–.58)	.43 (.14–.66)	.30 (−.02–.59)	.43 (.11–.69)
	14	.32 (.01–.59)	.34 (.01–.63)	.23 (−.11–.54)	.27 (−.06–.56)
	15	.70 (.51–.84)	.76 (.62–.83)	.61 (.35–.80)	.58 (.36–.76)
	16	.49 (.22–.71)	.51 (.29–.67)	.73 (.54–.86)	.56 (.28–.77)
	17	.54 (.23–.77)	.54 (.28–.71)	.63 (.39–.79)	.71 (.52–.84)
	18	.62 (.42–.76)	.59 (.36–.77)	.61 (.38–.78)	.57 (.33–.76)
Subjective quality	20	.94 (.90–.97)	.89 (.80–.94)	.89 (.79–.94)	.83 (.69–.90)
	21	.88 (.77–.94)	.86 (.75–.92)	.81 (.67–.89)	.81 (.69–.88)
	22	.88 (.81–.92)	.79 (.65–.86)	.81 (.65–.90)	.69 (.51–.80)
	23	.95 (.91–.97)	.94 (.89–.97)	.89 (.79–.94)	.88 (.79–.94)

^a^Item 19 was excluded from all calculations because of lack of ratings

Pearson’s correlation coefficients between the subscales making up the MARS total score (the objective subscales) are reported in Table 5. Only the subscales “engagement” and “aesthetics” showed *r* values above .7 for both raters. Of the 22 items considered, 20 (91 %) displayed a higher correlation with their own subscale than with other subscales (Additional file 2: Table S2).

**Table 5:** Between-subscale (objective subscales) Pearson’s *r* correlation coefficients, by rater (Rater 1: upper right triangle; Rater 2: lower left triangle)

Subscale	Engagement	Functionality	Aesthetics	Information
Engagement	–	.29 (−.01–.54)	.72 (.54–.84)	.61 (.38–.77)
Functionality	.34 (.04–.58)	–	.34 (.04–.58)	.53 (.27–.72)
Aesthetics	.77 (.61–.87)	.47 (.20–.68)	–	.43 (.15–.65)
Information^a^	.56 (.31–.74)	.66 (.45–.80)	.49 (.22–.69)	–

^a^ Item 19 was excluded from all calculations because of lack of ratings

Bootstrapped generalised Ferguson's *δ* coefficients ranged from .84 to .96 and from .86 to .96 for raters 1 and 2, respectively, indicating that the questionnaire is able to establish differences among the apps. As shown by Loevinger's *H* coefficients, the scalability of all the subscales and the MARS total score was acceptable, exceeding the threshold value of .3 (Additional file 2: Table S3).

Thirty-seven (86 %) apps had at least one vote in an app store; of these, 31 and 23 had at least 5 and 10 votes, respectively. As shown in Table 6, the number of votes for an app affected the strength of association between MARS items or subscales (MARS star rating, MARS total score and subjective quality subscales) and the star ratings available in the app stores: the more votes that were given, the more significant was the positive association observed, regardless of both rater and item/scales. However, on applying a 10-vote cut-off, the statistically significant correlation coefficient was only poor to moderate.

**Table 6:** Correlation coefficients between rating systems available in app stores and MARS star rating, total and subjective quality scores, by number of ratings cut-off and rater

MARS item/scale	N of apps (%)	N of ratings cut-off	Rater 1		Rater 2	
			Estimate	*p*	Estimate	*p*
MARS star rating (N23)^a^	37 (86.0)	1	.18	.28	.26	.12
	31 (72.1)	5	.25	.17	.31	.086
	23 (53.5)	10	.50	.015	.46	.028
MARS total score^b^	37 (86.0)	1	.02	.92	.09	.62
	31 (72.1)	5	.03	.89	.09	.62
	23 (53.5)	10	.43	.041	.37	.081
App subjective quality^b^	37 (86.0)	1	.16	.35	.20	.23
	31 (72.1)	5	.19	.30	.26	.16
	23 (53.5)	10	.50	.015	.54	.008

^a^ Spearman’s *ρ* correlation coefficient; ^b^ Pearson’s *r* correlation coefficient

The MARS total score of the apps developed by governmental or non-profit organizations or universities [3.83 (SD 0.47)] was significantly higher (*t* = 4.25, *p* < .001) than that [3.12 (SD 0.61)] of the apps from unknown/commercial developers. The effect size was large [*d* = 1.30 (95 % CI: 0.62–1.98)]. Similarly, comparison of single subscales (Additional file 2: Figure S2) revealed lower scores for the apps from unknown/commercial developers; the highest effect size of 1.61 (95 % CI: 0.90–2.32) was seen for the information subscale, while the lowest concerned aesthetics [*d* = 0.76 (95 % CI: 0.13–1.40)].

The internal consistency of the MARS total score was very similar between the Italian version and the original version [*α*s of .92 vs .90, respectively; *F* = 1.25, *p* = .45]. In our study, the ICC for the total score was substantially higher (.96 vs .79), with non-overlapping 95 % CIs. By contrast, the Australian version of MARS displayed higher concurrent validity with the app stores rating system, though the difference did not reach an *α* < .05 on applying either the 5-vote (*z* = 1.62, *p* = .11) or 10-vote (*z* = 0.53, *p* = .59) cut-off.

The model that best predicted the MARS total score consisted of two predictors, namely the app-store star rating and the developer’s affiliation. The former was, however, not statistically significant (*b* = 0.09, *p* = .48). By contrast, institutional (governmental, non-profit organization, university) affiliation was significantly (*p* < .001) associated with a 0.82 increase in the MARS total score. The model explained 39.8 % of variance.

## Discussion

Italian is one of the top 10 languages used on the web [43]. The increasing number of mHealth apps and smartphone owners in Italy makes it essential for professional users, providing they are appropriately trained, to have a standardised tool for assessing the quality of health-related mobile apps. The present study produced an Italian version of the MARS, and established its validity and reliability in assessing the quality of apps targeting primary prevention issues and available in different app stores. Moreover, this study is among the first to validate an English language mHealth assessment tool in another language. We can conclude that the Italian version of MARS is functionally equivalent to the source tool, as all the types of equivalence were achieved.

In our opinion, the Italian version of MARS can be used by any relevant stakeholders including, for example, public health authorities, patient organisations, healthcare professionals and app developers, the ultimate goal being to provide laypeople with high-quality apps. Our scale could be successfully applied by IT specialists; for instance, the English version of MARS has already been used by website developers who recommended apps on their pages by adding the total MARS score next to the app description. It is, however, recommended that raters attend a specific training course [18], which lasts no more than one hour, in order to calibrate their future ratings. The training free user version of the MARS (uMARS) has been recently validated and the results will be published soon. An Italian user version of MARS is now being studied; we will also evaluate MARS-based quality ratings given to a particular app by “real world” users.

Translating IT terminology, which is full of anglicisms, may be a challenge [44] in fields unrelated to IT, such as public health and healthcare. Unlike some other languages, Italian tends to absorb IT terminology in a non-adapted form. Before undertaking the first step of translation, we examined local websites dealing with mobile technology, including press releases from major smartphone producers, and noted several discrepancies in the translation of terms with the same semantic-referential meanings (some websites have tended to use non-adapted terminology, while others have worked for some degree of adaptation/integration). Critical appraisal of these semantic neologisms will help to achieve linguistic equivalence between source and target languages. Since steady technological progress will determine a rise in novel “necessity” borrowings in the growing mHealth field in Italy, prompt standardisation of IT terminology would be beneficial.

The psychometric properties of the Italian version of MARS were similar to those of the original English version of the scale. The internal consistency of the Italian version proved comparable to that of the original tool and fully complies with the internationally established quality criteria [28]. Inter-rater reliability ranged from good to excellent, thus confirming the initial findings [18] that MARS can be used with high confidence by single raters. Convergent validity, unidimensionality and discriminatory properties also proved satisfactory.

Although most pre-specified quality criteria were fulfilled, some validation parameters at the level of single subscales were not met (these may be regarded as study limitations). The functionality subscale showed a considerable ceiling effect; the mean score for this subscale was also higher than those of other subscales. This latter finding is in line with the patterns of summary scores (the greatest summary score of 4.0 for functionality) obtained by Stoyanov et al. [18]. We nevertheless believe that this result has very little impact on the validity of the scale for two reasons. First, the MARS functionality subscale is an objective subscale; indeed, each of the 5 possible levels of an item states clear requirements that an app must have in order to be assigned to that level (score). Second, in our study, several apps were purely informational and involved technically simple and basically efficient tasks, which made them easy to use and to navigate in; this probably explains the relatively high functionality score. Another interesting finding concerns the high correlation between aesthetics and engagement, which may suggest that these two objective subscales assess closely related constructs, although the estimates were not much above the arbitrary threshold of .7. This observation is, however, plausible, since a high level of visual appeal and high-quality graphics may enhance user engagement and experience [45]. For example, Stenalt and Godsk [46] found that 82 % of interviewees believed that design and layout play a crucial role in engagement with e-learning platforms.

Contrary to the original report [18], we failed to establish a significant correlation between the MARS total score and the app-store star ratings for apps with more than 5 votes (though we did when the cut-off was raised to 10 votes). However, the app-store star-rating system is highly subjective and can hardly be regarded as a reliable and objective measure of app quality [18]. We hypothesize that the discrepancy observed was due to “information asymmetry” between MARS raters and “real-world” app voters with regard to app quality attributes. Indeed, as underlined by Stoyanov et al. [18], app users’ ratings are based on scattered criteria of a subjective nature. The trustworthiness of apps with few ratings may also be compromised by fake reviews from app developers [47]; this may partly explain why the strength of correlation between real users’ ratings and the MARS total score increased as the cut-off number of ratings increased. However, this latter finding should be interpreted cautiously, as the study had a different objective. Indeed, the sample size was estimated in order to establish inter-rater reliability, and therefore a relatively small number of apps were used in the analysis. In any case, we demonstrated that all the MARS summary scores displayed known-groups validity, which is a type of concurrent validity.

Other possible limitations should be mentioned. First, most “foreign-born” apps were poorly translated into Italian. We acknowledge that this fact could affect our ratings, especially those regarding the information subscale. Second, we saw that the app store descriptions might not be accurate (the average between-rater score on item 13 on the accuracy of app descriptions in app stores was in the range of 1–5). Therefore, some apps available in Italy and relevant to primary prevention may have been discarded. App developers should provide a detailed description of app features and functionalities, in order to better inform potential users. Similarly, most of our prevention-related search terms (such as *prevention*) were quite “general”; in such cases, for instance, Google Play yields a maximum of 250 apps and, therefore, some relevant lower-ranked apps may have been omitted. This limitation has also been previously reported [25]. In future research, it will be useful to select apps in a systematic way from a publicly available database. In this regard, for example, Xu and Liu [48] have created a repository of health-related apps with more than 60,000 entries. However, this database contains only apps from US, Chinese, Japanese, Brazilian and Russian app stores. Finally, as in previous studies [18, 19], item 19 “evidence based” was excluded from all statistical calculations because of the lack of ratings. Nevertheless, we believe that this item would have shown good psychometric properties, as it among the most “objective” MARS items: it can easily be verified in scientific databases such as Google Scholar. As proof of this, both evaluators were able to locate the sole rateable app and attributed the same score.

## Conclusions

Although the original version of MARS was designed for mHealth experts and researchers, a simplified training-free version of the scale to obtain app-user quality ratings will be available soon. Expert and app-user MARS ratings of primary prevention apps could benefit general practitioners (GPs) and other healthcare professionals by providing clear guidelines on which high-quality apps in the preventive medicine and health promotion fields to recommend to their patients. This may also help to reduce barriers to preventive health counselling in general practice, including lack of time, knowledge, reimbursement and patient compliance [49–51]. A recent paper by Mani et al. [19], for example, recommends using a cut-off of 3.0 (60 %) as a minimum acceptable MARS total score. In conclusion, the Italian version of MARS provides trained researchers, mHealth specialists and health professionals with a valid and reliable measure of health app quality.

## Abbreviations

CBT, cognitive behavioral therapy; CI, confidence interval; GP, general practitioner; IT, information technology; MARS, mobile application rating scale; mHealth, mobile health; SD, standard deviation

## Additional files

Additional file 1: Mobile Application Rating Scale (versione italiana). This file contains the Italian version of MARS (versione italiana della *Mobile Application Rating Scale*). (PDF 277 kb) Additional file 2: Additional psychometric properties. This file reports intra-class, inter-item, item-subscale correlation coefficients, generalised Ferguson’s *δ* and Loevinger’s *H* scalability coefficients and Cohen’s *d*s of subscale score differences. (PDF 324 kb)
