# Supplementary material for: Development and validation of the Italian version of the Mobile Application Rating Scale and its generalisability to apps targeting primary prevention
Source: BMC Med Inform Decis Mak. 2016 Jul 7;16:83. doi: 10.1186/s12911-016-0323-2 (PMC4936279; doi:10.1186/s12911-016-0323-2)
Supplement: Additional file 1: — Mobile Application Rating Scale (versione italiana). This file contains the Italian version of MARS (versione italiana della Mobile Application Rating Scale). (PDF 277 kb) [file 12911_2016_323_MOESM1_ESM.pdf]

Mobile Application Rating Scale (versione italiana)

Classificazione della app

La sezione di classificazione è usata per raccogliere descrizione e informazioni tecniche sulla app. Visualizzi la descrizione della app su iTunes/Google Play/Windows Store per avere queste informazioni.

Nome app: ____

Voto app versione corrente: ____

Voto app tutte le versioni: ____

Sviluppatore: ____

N voti app versione corrente: ____

N voti app tutte le versioni: ____

Versione corrente: ____

Ultimo aggiornamento:____

Costo versione base: ____

Costo versione *upgrade*: ____

Piattaforma:

- ☐ iPhone
- ☐ iPad
- ☐ Android
- ☐ Windows Phone
- ☐ Altro _________________________________

Breve descrizione:

____

____

____

____

____

Obiettivo della app (più risposte possibili)

- ☐ Aumentare il senso di benessere
- ☐ *Mindfulness* (consapevolezza dei propri pensieri, azioni e motivazioni)/meditazione/rilassamento
- ☐ Ridurre emozioni negative
- ☐ Depressione
- ☐ Ansia/stress
- ☐ Rabbia
- ☐ Modifica del comportamento
- ☐ Alcol/uso di sostanze
- ☐ Fissare obiettivi
- ☐ Intrattenimento
- ☐ Relazioni
- ☐ Salute fisica
- ☐ Altro____

Base teorica/strategie (più risposte possibili)

- ☐ Valutazione
- ☐ Feedback
- ☐ Informazione/educazione
- ☐ Monitoraggio/rilevamento nel tempo
- ☐ Fissare obiettivi
- ☐ Consigli/suggerimenti/strategie/formazione di competenze
- ☐ TCC – Terapia cognitivo-comportamentale
- ☐ ACT - *Acceptance and Commitment Therapy* (Terapia di accettazione e di impegno nell'azione)
- ☐ *Mindfulness* (consapevolezza dei propri pensieri, azioni e motivazioni)/meditazione
- ☐ Rilassamento
- ☐ Gratitudine
- ☐ Approccio dei punti di forza
- ☐ Altro ______________________________________

Affiliazione (fonte):

- ☐ Sconosciuta
- ☐ Commerciale
- ☐ Governativa/ente pubblico
- ☐ ONG (Organizzazioni non governative)
- ☐ Università

Fasce di età (più risposte possibili)

- ☐ Bambini (sotto i 12 anni)
- ☐ Adolescenti (13-17 anni)
- ☐ Giovani adulti (18-25 anni)
- ☐ Adulti
- ☐ Generale (tutte le età)

Aspetti tecnici della app (più risposte possibili)

- ☐ Permette la condivisione (Facebook, Twitter ecc.)
- ☐ Ha una *community* (comunità virtuale/online) dedicata
- ☐ Permette la protezione tramite una password
- ☐ Richiede il login (credenziali)
- ☐ Invia dei *reminder* (promemoria)
- ☐ Per funzionare necessita della connessione Internet

Valutazione di qualità della app

La scala di valutazione misura la qualità della app in quattro dimensioni o scale. Tutti gli item (domande) vengono valutati su una scala a 5 punti che va da "1. Inadeguato" a "5. Eccellente". Scelga il numero che rappresenta in modo più accurato possibile la qualità dell'aspetto della app che Lei sta valutando. Usi le definizioni fornite per ciascuna categoria.

Sezione A.

Coinvolgimento – divertente, interessante, personalizzabile, interattiva (per esempio, invia notifiche, messaggi, promemoria, feedback, permette la condivisione), ben diretta al suo pubblico

1. **Intrattenimento: la app è divertente da usare? La app utilizza qualche strategia di intrattenimento (per esempio, attraverso la *gamification*/ludicizzazione) per aumentare il coinvolgimento?**
   1 Noiosa, non è per niente divertente
   2 Per lo più noiosa
   3 OK, sufficientemente divertente per intrattenere l'utente per un breve periodo di tempo (< 5 minuti)
   4 Abbastanza divertente, potrebbe intrattenere l'utente per un po' di tempo (5–10 minuti in totale)
   5 Molto divertente, potrebbe stimolare l'utente ad utilizzarla più volte

2. **Interesse: la app è interessante da usare? La app utilizza qualche strategia per aumentare il coinvolgimento presentando i suoi contenuti in modo interessante?**
   1 Non è per niente interessante
   2 Per lo più non interessante
   3 OK, né molto né poco interessante, potrebbe coinvolgere l'utente per un breve periodo di tempo (< 5 minuti)
   4 Abbastanza interessante, potrebbe coinvolgere l'utente per un po' di tempo (5–10 minuti in totale)
   5 Molto interessante, potrebbe stimolare l'utente ad utilizzarla più volte

3. **Personalizzazione: la app fornisce/memorizza tutte le impostazioni/preferenze necessarie per personalizzarne le caratteristiche (per esempio, suoni, contenuto, notifiche ecc.)?**
   1 Non permette alcuna personalizzazione o richiede che le impostazioni vengano inserite ogni volta
   2 Consente una insufficiente personalizzazione limitandone le funzioni
   3 Consente una personalizzazione di base per funzionare adeguatamente
   4 Fornisce numerose opzioni per la personalizzazione
   5 Consente una completa personalizzazione delle caratteristiche/preferenze, memorizza tutte le impostazioni

4. **Interattività: la app permette l'input da parte dell'utente, fornisce feedback, contiene suggerimenti (remainder/promemoria, opzioni di condivisione, notifiche, ecc.)? Nota: queste funzionalità devono essere personalizzabili e non sopraffare l'utente per essere perfette.**
   1 Nessuna funzionalità interattiva e/o nessuna risposta all'interazione con l'utente
   2 Interattività o feedback o possibilità di input dell'utente insufficienti, limitando le funzioni della app
   3 Funzionalità interattive di base per funzionare adeguatamente
   4 Offre una varietà di opzioni di funzionalità interattive/feedback/input dell'utente
   5 Livello di responsività molto elevato attraverso opzioni di funzionalità interattive/feedback/input dell'utente

5. **Gruppo target: il contenuto della app (informazioni visive, linguaggio, design) è appropriato per il pubblico a cui la app è destinata?**
   1 Completamente inappropriata/non chiara/genera sempre confusione
   2 Per lo più inappropriata/non chiara/genera confusione
   3 Accettabile ma non mirata. Potrebbe risultare inappropriata/non chiara/generare confusione
   4 Ben diretta all'obiettivo, con problemi trascurabili
   5 Perfettamente diretta all'obiettivo, priva di alcun problema

A. Punteggio medio del coinvolgimento = ________________

SEZIONE B

Funzionalità – funzionamento della app, facilità di apprendimento, navigazione, flusso logico e design d'interazione gestuale della app

6. **Prestazione: quanto sono accurati/veloci le funzioni e i componenti (pulsanti/menu) della app?**
   1 La app non funziona; risposta assente/insufficiente/inaccurata (per esempio, *crash*: blocchi o terminazioni improvvise, *bug*: difetti di programmazione, comandi non funzionanti ecc.)
   2 Alcune funzionalità vanno ma sono lente o contengono problemi tecnici maggiori
   3 A grandi linee la app funziona. A volte, ci sono alcuni problemi tecnici da risolvere/rallentamenti
   4 Per lo più funzionale con problemi minori/trascurabili
   5 Risposta perfetta/tempestiva; nessun *bug* tecnico (difetti di programmazione)/contiene un misuratore di "tempo di caricamento rimanente"

7. **Facilità di utilizzo: quanto è facile imparare a usare la app; quanto sono chiare le etichette/icone del menu e le istruzioni?**
   1 Istruzioni mancanti/limitate; le etichette/icone del menu generano confusione; complicata
   2 Usabile dopo molto tempo/molti sforzi
   3 Usabile dopo qualche tempo/qualche sforzo
   4 Facile da imparare (o contiene istruzioni chiare)
   5 Si usa immediatamente; intuitiva; semplice

8. **Navigazione: il muoversi tra le schermate è logico/accurato/appropriato/ininterrotto; i link/collegamenti a tutte le schermate che li necessitano sono presenti?**
   1 All'interno della app diverse sezioni sembrano logicamente scollegate e la navigazione è difficile
   2 Usabile dopo molto tempo/molti sforzi
   3 Usabile dopo qualche tempo/qualche sforzo
   4 Facile da usare o mancante di pochi trascurabili link/collegamenti
   5 Il flusso tra le schermate è sempre perfettamente logico, facile, chiaro e intuitivo o offre scorciatoie

9. **Design d'interazione gestuale: le interazioni tramite tocco (per esempio, *tap*: toccare, *swipe*: scorrere in orizzontale, *scroll*: scorrere in verticale, *pinch*: zoomare) sono coerenti e intuitive in tutti i componenti e in tutte le schermate?**
   1 Completamente incoerente/genera sempre confusione
   2 Spesso incoerente/genera spesso confusione
   3 OK con qualche elemento incoerente/talvolta genera confusione
   4 Per lo più coerente/intuitiva con problemi trascurabili
   5 Perfettamente coerente e intuitiva

B. Punteggio medio della funzionalità = ________________

SEZIONE C

Estetica – design grafico, piacevolezza visiva complessiva, schema colore e coerenza stilistica

10. **Layout (disposizione): la collocazione e le dimensioni di pulsanti/icone/menu/contenuti sulle schermate sono appropriate o possono essere ingrandite/zoomate se necessario?**
    1 Design molto scarso, caotico, alcune opzioni impossibili da selezionare/localizzare/vedere/leggere; schermo del dispositivo non ottimizzabile
    2 Design scarso, casuale, non chiaro, alcune opzioni difficili da selezionare/localizzare/vedere/leggere
    3 Soddisfacente, pochi problemi nel selezionare/localizzare/vedere/leggere le opzioni o con problemi minori di dimensione dello schermo
    4 Per lo più chiaro, permette di selezionare/localizzare/vedere/leggere le opzioni
    5 Professionale, semplice, chiaro, ordinato, organizzato in modo logico, ottimizzato alle dimensioni dello schermo del dispositivo. Ogni componente del design ha il suo scopo

11. **Grafica: quanto è alta la qualità/risoluzione della grafica usata per pulsanti/icone/menu/contenuti?**
    1 La grafica appare amatoriale, design visivo molto scarso: sproporzionato, completamente incoerente dal punto di vista stilistico
    2 Grafica di bassa qualità/bassa risoluzione; design visivo di bassa qualità: sproporzionato, incoerente dal punto di vista stilistico
    3 Grafica e design visivo di media qualità (per lo più coerente nello stile)
    4 Grafica e design visivo di alta qualità e risoluzione: per lo più proporzionato, coerente dal punto di vista stilistico
    5 Grafica e design visivo di altissima qualità e risoluzione: proporzionato, coerente dal punto di vista stilistico

12. **Piacevolezza visiva: la app è bella?**
    1 Nessuna piacevolezza visiva, sgradevole da guardare, mal disegnata, colori mal abbinati
    2 Poca piacevolezza visiva: mal disegnata, cattivo uso dei colori, visivamente noiosa
    3 Media piacevolezza visiva: ordinaria, né gradevole né sgradevole
    4 Alto livello di piacevolezza visiva: grafica senza stonature, coerente e professionalmente disegnata
    5 Come sopra + molto attraente, da ricordare, degna di nota; l’uso dei colori potenzia le funzionalità/menu della app

C. Punteggio medio dell’estetica = ________________

SEZIONE D

Informazioni – contiene informazioni di alta qualità (per esempio, testo, feedback, misure, bibliografia) provenienti da fonti credibili. Selezioni N/A se quell'aspetto della app è irrilevante

13. Accuratezza della descrizione della app (nell'app store): la app contiene ciò che è descritto?

1 Fuorviante. La app non contiene i componenti/le funzionalità descritti. Oppure non ha descrizione
2 Inaccurata. La app contiene pochissimi dei componenti/funzionalità descritti
3 OK. La app contiene alcuni dei componenti/funzionalità descritti
4 Accurata. La app contiene la maggior parte dei componenti/funzionalità descritti
5 Descrizione molto accurata dei componenti/funzionalità della app

14. Obiettivi: la app ha obiettivi specifici, misurabili e raggiungibili (specificati nella descrizione dell'app store o all'interno della app)?

N/A La descrizione non indica gli obiettivi o gli obiettivi della app sono irrilevanti allo scopo della ricerca (per esempio, un gioco per scopi educativi)
1 La app non ha possibilità di raggiungere gli obiettivi prefissati
2 La descrizione elenca alcuni obiettivi ma la app ha pochissima possibilità di raggiungerli
3 OK. La app ha obiettivi chiari, che possono essere raggiunti
4 La app ha obiettivi chiaramente specificati, che sono misurabili e raggiungibili
5 La app ha obiettivi specifici e misurabili, che molto probabilmente saranno raggiunti

15. Qualità delle informazioni: il contenuto della app è corretto, ben scritto e rilevante per l'obiettivo/argomento della app?

N/A Non ci sono informazioni all'interno della app
1 Irrilevante/inappropriato/incoerente/scorretto
2 Scarso. A malapena rilevante/appropriato/coerente/può essere scorretto
3 Mediamente rilevante/appropriato/coerente/sembra corretto
4 Rilevante/appropriato/coerente/corretto
5 Altamente rilevante, appropriato, coerente e corretto

16. Quantità di informazioni: è sufficiente per raggiungere l'obiettivo della app; le informazioni sono complete ma sintetiche?

N/A Non ci sono informazioni all'interno della app
1 Minima o eccessiva
2 Insufficiente o verosimilmente eccessiva
3 OK ma non completa né sintetica
4 Offre un ampio spettro di informazioni, presenta alcune lacune o inutili dettagli o non ha link/collegamenti a ulteriori informazioni o risorse
5 Completa e sintetica; contiene link/collegamenti a ulteriori informazioni o risorse

17. Informazioni visuali: la spiegazione visiva dei concetti attraverso diagrammi/grafici/immagini/video ecc. è chiara, logica, corretta?

N/A Non ci sono informazioni visuali all'interno della app (per esempio, contiene solo audio o testo)
1 Completamente non chiare/generano sempre confusione/sbagliate o necessarie ma mancanti
2 Per lo più non chiare/generano confusione/sbagliate
3 OK ma spesso non chiare/generano confusione/sbagliate
4 Per lo più chiare/logiche/corrette con problemi trascurabili
5 Perfettamente chiare/logiche/corrette

18. **Credibilità: la app proviene da una fonte attendibile (specificata nella descrizione dell'app store o all'interno della app)?**
    1 La fonte si identifica ma la sua credibilità/attendibilità è dubbia (per esempio, attività commerciale con tornaconto personale)
    2 Sembra avere una fonte credibile che però non può essere verificata (per esempio, non ha una pagina web)
    3 Sviluppata da una piccola organizzazione non governativa/istituzione (ospedale/centro ecc.)/attività commerciale specializzata, agenzia di finanziamento
    4 Sviluppata dal governo, dall'università o come sopra ma di dimensioni maggiori
    5 Sviluppata utilizzando finanziamenti governativi su base competitiva o fondi di ricerca (per esempio, MIUR – Ministero dell'Istruzione, dell'Università e della Ricerca)

19. **Prove di efficacia: la app è stata testata/studiata; deve essere verificabile (pubblicazioni nella letteratura scientifica)?**
    N/A La app non è stata testata/studiata
    1 L'evidenza suggerisce che la app non funziona
    2 La app è stata studiata (per esempio, accettabilità, usabilità, valutazione di soddisfazione) e ha outcomes parzialmente positivi in studi non randomizzati controllati o poche prove di efficacia o assenza di prove di efficacia contraddittorie
    3 La app è stata studiata (per esempio, accettabilità, usabilità, valutazione di soddisfazione) e ha outcomes positivi in studi non randomizzati controllati e assenza di prove di efficacia contraddittorie
    4 La app è stata studiata in 1-2 studi randomizzati controllati che hanno verificato l'outcome con risultati favorevoli
    5 La app è stata studiata in ≥ 3 studi randomizzati controllati che hanno verificato l'outcome con risultati favorevoli

D. Punteggio medio delle informazioni = ________________*

*Escluda le domande la cui risposta è N/A dal calcolo della media

Qualità soggettiva della app

Sezione E

20. Raccomanderebbe questa app alle persone che potrebbero beneficiarne?

1	Assolutamente no	Non raccomanderei questa app a nessuno
2		Raccomanderei questa app a poche persone
3	Forse	Raccomanderei questa app a diverse persone
4		Raccomanderei questa app a molte persone
5	Assolutamente sì	Raccomanderei questa app a tutti

21. Quante volte utilizzerebbe questa app nei prossimi 12 mesi se fosse utile per Lei?

1 Nessuna
2 1-2
3 3-10
4 10-50
5 > 50

22. Comprerebbe questa app?

1 No
3 Forse
5 Sì

23. Come valuta complessivamente la app?

1	★	Una delle peggiori app mai usate
2	★★	
3	★★★	Nella media
4	★★★★	
5	★★★★★	Una delle migliori app mai usate

Punteggio

Punteggi di qualità della app per

SEZIONE

A. Punteggio medio del coinvolgimento = ________________

B. Punteggio medio della funzionalità = ________________

C. Punteggio medio dell’estetica = ________________

D. Punteggio medio delle informazioni = ________________

Punteggio medio qualità della app = ________________

Punteggio qualità soggettiva della app = ________________

Sezione specifica per la app

Questi item supplementari possono essere modificati e usati per valutare l'impatto percepito della app su conoscenze, atteggiamenti, intenzioni di cambiare dell'utente così come la probabilità di un'effettiva modifica del comportamento relativo alla salute in oggetto.

Sezione F

1. **Consapevolezza: è probabile che questa app aumenti la consapevolezza dell'importanza di affrontare [inserire il comportamento relativo alla salute in oggetto]**
   1 In completo disaccordo
   2
   3
   4
   5 Completamente d'accordo

2. **Conoscenze: è probabile che questa app aumenti le conoscenze/comprensione di [inserire il comportamento relativo alla salute in oggetto]**
   1 In completo disaccordo
   2
   3
   4
   5 Completamente d'accordo

3. **Atteggiamenti: è probabile che questa app migliori gli atteggiamenti relativamente a [inserire il comportamento relativo alla salute in oggetto]**
   1 In completo disaccordo
   2
   3
   4
   5 Completamente d'accordo

4. **Intenzioni di cambiare: è probabile che questa app aumenti le intenzioni/motivazioni di affrontare [inserire il comportamento relativo alla salute in oggetto]**
   1 In completo disaccordo
   2
   3
   4
   5 Completamente d'accordo

5. **Richiesta di aiuto (*help seeking*): è probabile che l'uso di questa app incoraggi ulteriormente la richiesta di aiuto per [inserire il comportamento relativo alla salute in oggetto]**
   1 In completo disaccordo
   2
   3
   4
   5 Completamente d'accordo

6. **Modifica del comportamento: è probabile che l'uso di questa app aumenti/diminuisca [inserire il comportamento relativo alla salute in oggetto]**
   1 In completo disaccordo
   2
   3
   4
   5 Completamente d'accordo
