# Supplementary material for: Development and validation of the Italian version of the Mobile Application Rating Scale and its generalisability to apps targeting primary prevention
Source: BMC Med Inform Decis Mak. 2016 Jul 7;16:83. doi: 10.1186/s12911-016-0323-2 (PMC4936279; doi:10.1186/s12911-016-0323-2)

Table S1 Intra-class correlation coefficients, by item

Subscale	Item	ICC		
		Estimate	95% CI	*p*
Engagement	1	.90	.81 – .95	<.001
	2	.86	.74 – .93	<.001
	3	.88	.77 – .93	<.001
	4	.74	.52 – .86	<.001
	5	.60	.27 – .78	.001
Functionality	6	.90	.82 – .95	<.001
	7	.76	.57 – .87	<.001
	8	.75	.54 – .87	<.001
	9	.65	.35 – .81	<.001
Aesthetics	10	.89	.78 – .94	<.001
	11	.84	.70 – .91	<.001
	12	.88	.79 – .94	<.001
Information*	13	.88	.77 – .93	<.001
	14	.72	.47 – .85	<.001
	15	.92	.85 – .96	<.001
	16	.87	.75 – .93	<.001
	17	.59	.18 – .80	.006
	18	.92	.86 – .96	<.001
Subjective quality	20	.93	.87 – .96	<.001
	21	.90	.81 – .95	<.001
	22	.82	.61 – .91	<.001
	23	.93	.81 – .97	<.001

*Item 19 was excluded from all calculations because of lack of ratings

Fig. S1 Spearman's ρ inter-item correlation coefficients, by rater (Rater 1: upper right triangle; Rater 2: lower left triangle) and subscale

**i1**	.89	.35	.52	.60	.12	.13	.20	.31	.76	.66	.73	.13	.08	.37	.62	.54	.53	.70	.68	.62	.75
.85	**i2**	.43	.54	.59	.23	.18	.22	.31	.63	.61	.69	.08	.14	.42	.65	.53	.55	.78	.76	.69	.86
.69	.65	**i3**	.51	.26	-.03	-.32	-.16	.03	.28	.44	.38	.07	-.20	.10	.40	.21	.24	.39	.53	.50	.40
.39	.41	.34	**i4**	.47	.15	-.05	-.04	.26	.57	.39	.40	.14	.16	.32	.58	.27	.34	.52	.55	.56	.55
.52	.51	.44	.28	**i5**	.53	.25	.51	.51	.57	.41	.43	.35	.24	.61	.60	.48	.60	.77	.66	.69	.71
.07	.11	.07	-.01	.12	**i6**	.32	.64	.58	.26	.10	.18	.38	.43	.54	.17	.32	.29	.47	.39	.40	.43
.32	.42	.18	-.04	.34	.51	**i7**	.56	.42	.31	-.12	.03	.00	.16	.40	.28	.21	.36	.33	.10	.17	.30
.45	.51	.39	.17	.33	.63	.60	**i8**	.53	.26	.06	.18	.23	.27	.39	.21	.12	.38	.42	.25	.24	.34
.41	.41	.32	.11	.41	.55	.79	.74	**i9**	.46	.21	.28	.22	.11	.31	.16	.20	.38	.46	.26	.36	.39
.54	.47	.30	.15	.53	.38	.55	.46	.63	**i10**	.63	.73	.23	.14	.40	.66	.61	.47	.64	.57	.64	.65
.77	.70	.61	.23	.66	.10	.35	.42	.42	.60	**i11**	.82	.01	-.11	.18	.50	.33	.19	.45	.53	.49	.52
.77	.73	.61	.20	.58	.10	.31	.44	.40	.58	.92	**i12**	.00	.00	.14	.49	.41	.27	.51	.58	.62	.60
.20	.35	.31	.38	.32	.48	.36	.46	.32	.20	.19	.13	**i13**	.43	.22	.24	.31	.18	.24	.16	.14	.18
-.01	.17	.11	.31	.24	.57	.25	.39	.21	.16	.02	.02	.65	**i14**	.30	.14	.38	.24	.30	.33	.21	.30
.33	.35	.34	.11	.41	.45	.28	.44	.37	.33	.34	.39	.30	.24	**i15**	.54	.57	.73	.74	.58	.56	.68
.41	.45	.30	.23	.45	.26	.35	.42	.48	.42	.36	.41	.16	.02	.57	**i16**	.34	.51	.68	.64	.62	.73
.50	.51	.41	.01	.53	.34	.46	.49	.50	.60	.70	.71	.31	.19	.54	.66	**i17**	.41	.66	.50	.61	.62
.36	.35	.36	.19	.41	.36	.31	.45	.43	.38	.24	.34	.25	.30	.69	.39	.23	**i18**	.79	.66	.54	.72
.58	.61	.47	.23	.64	.37	.53	.51	.56	.61	.61	.63	.26	.27	.72	.66	.76	.58	**i20**	.88	.85	.95
.68	.68	.47	.18	.59	.31	.54	.51	.48	.65	.68	.74	.32	.18	.58	.49	.75	.49	.79	**i21**	.81	.87
.57	.63	.42	.17	.61	.18	.37	.40	.26	.46	.64	.67	.29	.25	.50	.39	.63	.45	.76	.72	**i22**	.85
.71	.74	.55	.35	.67	.32	.50	.59	.53	.62	.75	.76	.30	.25	.70	.67	.76	.55	.91	.87	.76	**i23**

Spearman's $\rho \geq .2$ Spearman's $\rho < .2$ Engagement subscale   Functionality subscale

Aesthetics subscale   Information subscale   Subjective quality subscale

Table S2 Item-subscale (uncorrected) Spearman's ρ correlation coefficients, by rater

Subscale	Item	Rater 1					Rater 2				
		ENG	FUN	AES	INF*	SUB	ENG	FUN	AES	INF*	SUB
ENG	1	**.90**	.24	.82	.54	.73	**.89**	.37	.77	.43	.67
	2	**.91**	.31	.74	.58	.81	**.89**	.44	.71	.52	.71
	3	**.58**	-.11	.42	.19	.45	**.81**	.31	.59	.46	.51
	4	**.74**	.12	.48	.46	.59	**.61**	.09	.18	.33	.25
	5	.74	.57	.52	.72	**.76**	.67	.32	.66	.57	**.68**
FUN	6	.26	**.81**	.21	.51	.47	.11	**.78**	.20	.56	.37
	7	.08	**.71**	.05	.41	.26	.33	**.84**	.42	.46	.55
	8	.21	**.84**	.19	.42	.38	.49	**.87**	.53	.64	.56
	9	.37	**.80**	.34	.39	.42	.43	**.89**	.53	.57	.54
AES	10	.71	.42	**.85**	.62	.68	.51	.58	**.75**	.50	.65
	11	.62	.11	**.90**	.28	.53	.76	.37	**.95**	.42	.71
	12	.68	.24	**.93**	.33	.60	.75	.36	**.95**	.45	.75
INF*	13	.20	.27	.08	**.52**	.21	.38	.48	.19	**.60**	.32
	14	.15	.31	.02	**.53**	.32	.21	.41	.06	**.52**	.27
	15	.46	.50	.29	**.83**	.71	.40	.40	.42	**.83**	.70
	16	.70	.27	.58	.71	**.72**	.46	.41	.43	**.70**	.63
	17	.53	.29	.50	**.67**	.64	.52	.51	.74	.69	**.80**
	18	.60	.43	.35	**.77**	.74	.44	.41	.38	**.72**	.58
SUB	20	.81	.54	.60	.85	**.97**	.65	.55	.67	.79	**.95**
	21	.81	.35	.64	.69	**.92**	.68	.52	.78	.66	**.92**
	22	.77	.39	.66	.67	**.91**	.62	.32	.67	.59	**.82**
	23	.85	.47	.66	.80	**.97**	.78	.55	.80	.78	**.97**

The highest ρs within subscales are highlighted in bold.

ENG, engagement; FUN, functionality; AES, aesthetics; INF, information; SUB, subjective quality.

*Item 19 was excluded from all calculations because of lack of ratings

Table S3 Generalised Ferguson's δ and Loevinger's H scalability coefficients, by rater and subscale

Subscale	Ferguson's δ *(95% CI)*		Loevinger's H (SE)	
	Rater 1	Rater 2	Rater 1	Rater 2
Engagement	.91 (.88 – .93)	.90 (.86 – .92)	.62 (.07)	.62 (.07)
Functionality	.84 (.79 – .88)	.86 (.82 – .89)	.55 (.09)	.73 (.06)
Aesthetics	.86 (.80 – .89)	.86 (.80 – .90)	.87 (.05)	.82 (.06)
Information*	.93 (.91 – .95)	.92 (.89 – .95)	.44 (.08)	.44 (.09)
MARS total score	.96 (.95 – .97)	.96 (.95 – .97)	.38 (.06)	.45 (.07)
Subjective quality	.88 (.82 – .92)	.89 (.85 – .91)	.95 (.02)	.95 (.02)

*Item 19 was excluded from all calculations because of lack of ratings

Fig. S2 Cohen's *d*s of score differences (average of two raters) in the MARS subscales

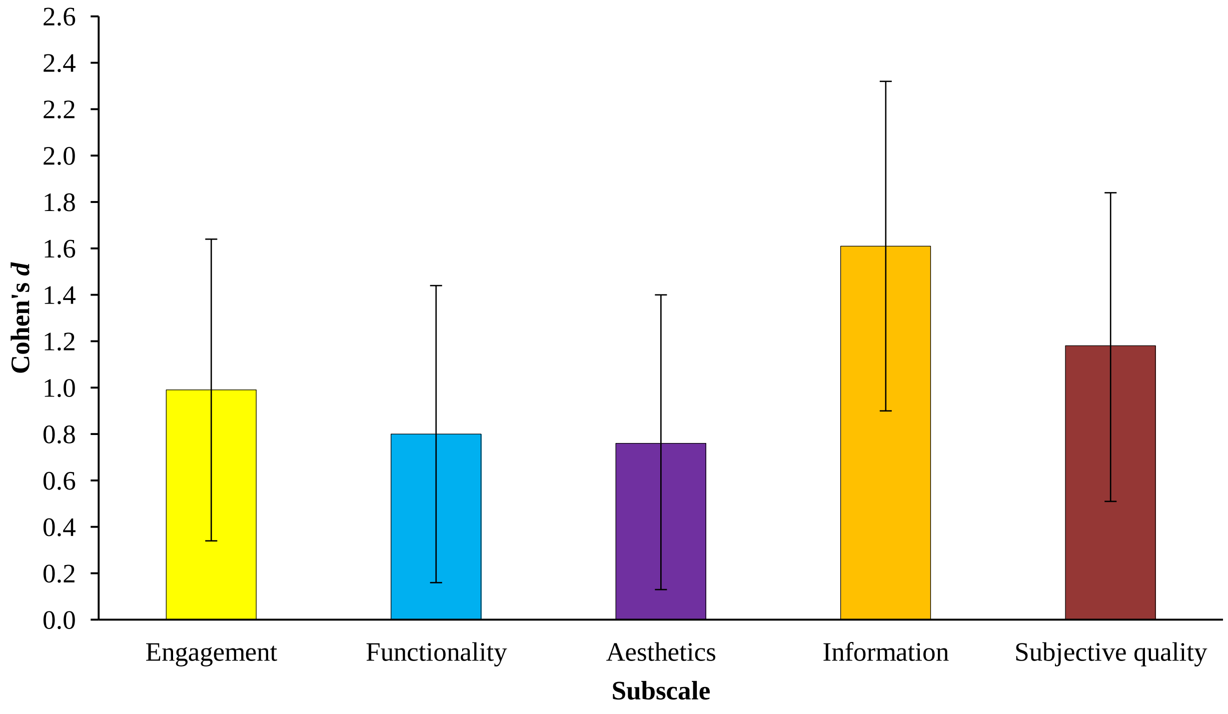

Supplement: Additional file 2: — Additional psychometric properties. This file reports intra-class, inter-item, item-subscale correlation coefficients, generalised Ferguson’s δ and Loevinger’s H scalability coefficients and Cohen’s ds of subscale score differences. (PDF 324 kb) [file 12911_2016_323_MOESM2_ESM.pdf]
